# Statin treatment for cerebral small vessel disease: A systematic review and meta-analysis of randomized controlled trials

**DOI:** 10.1016/j.cccb.2025.100389

**Published:** 2025-06-29

**Authors:** David Fresnais, Brynjar Fure

**Affiliations:** a School of Medical Sciences, Faculty of Medicine and Health, Örebro University, Örebro, Sweden; b Department of Internal Medicine, Central Hospital Karlstad, Karlstad, Sweden

**Keywords:** Dementia, Cognitive impairment, Statins, Cerebral small vessel disease, Meta-analysis

## Abstract

**Background:**

Cerebral small vessel disease (CSVD) is a chronic, progressive disorder that affects small blood vessels in the brain's white matter. This white matter damage appears on brain imaging as white matter hyperintensities (WMH). Although CSVD is often asymptomatic, it causes one fifth of strokes and nearly half of all vascular dementia cases, highlighting its clinical importance. Statins, widely used to lower lipid levels, are effective for reducing cardiovascular mortality in high-risk groups and are recommended for patients with myocardial infarction and ischemic stroke.

**Aim:**

This systematic review and meta-analysis of randomized controlled trials aimed to evaluate the impact of statin therapy on CSVD.

**Method:**

A literature search was performed in PubMed, Embase, the Cochrane Library and Epistemonikos resulting in the full-text review of 86 articles, of which two were used for the meta-analysis.

**Results:**

A non-significant trend towards lower WMH volume in milliliters was observed in the statin group compared to controls, mean difference (MD) = -4.44 (95 % CI -10.19–1.31).

**Conclusion:**

There is limited evidence available for the use of stain-treatment for CSVD, and further research is needed as well as studies on the clinical and person-centered benefits of statins on cognition and functional level in persons with CSVD.

## Highlights

• Statins may reduce white matter hyperintensities on brain imaging although current evidence is limited.
• The mechanisms behind the possible effect of statins for cerebral small vessel disease are not yet known.
• Future studies are needed and should focus on patient-centered and clinical outcomes and not exclusively on radiological outcomes.

## 1 Introduction

Cerebral small vessel disease (CSVD) is a chronic and progressive condition affecting the small vessels and capillaries supplying the brain’s white matter and subcortical structures, where the damage can be visualized as white matter hyperintensities (WMH), lacunes, enlarged perivascular spaces and microbleeds on brain magnetic resonance imaging (MRI) or computed tomography [1].

The prevalence of CSVD increases with age, affecting approximately 5 % of individuals in their 50 s and nearly 100 % of individuals in their 90 s [2]. The most common cause of CSVD is sporadic and associated with age and cardiovascular risk factors, especially hypertension [3]. In the 1960s, *Fisher* performed autopsies on patients with lacunar stroke and described pathological changes affecting small vessels less than 1 mm in diameter; *lipohyalinosis*, in which the accumulation of lipids leads to vessel wall-thickening and narrowing of lumen, and *microatheroma* [4]. These processes, along with arteriolosclerosis, are considered the most important pathophysiological mechanisms in hypertension-related CSVD. Other causes of CSVD include cerebral amyloid angiopathy, inflammatory and immune-mediated vascular diseases, and cerebral autosomal dominant arteriopathy with subcortical infarcts and leukoencephalopathy (CADASIL) [3].

CSVD is often asymptomatic, yet it accounts for 20 % of strokes and nearly half of vascular dementia cases, making it a clinically significant problem. Currently, no targeted treatments for CSVD exist beyond the modification of cardiovascular risk factors such as hypertension, hyperlipidemia, and diabetes mellitus [1]. Statins, which are the most commonly used drugs for treating hyperlipidemia, have been shown to reduce cardiovascular mortality in at-risk populations and are recommended by current guidelines for patients with both myocardial infarction and ischemic stroke [5,6]. Some studies have suggested that statins may reduce WMH burden in CSVD [7,8], while others have not demonstrated a clear effect [9].

Our understanding of the pathophysiological processes underlying CSVD remains limited, and although the pathophysiological processes involve deposition of lipids in intracerebral small vessels, the benefit of lipid-lowering treatments in CSVD is debated [10]. Therefore, the present meta-analysis was performed to investigate whether statins can affect the progression of CSVD.

## 2 Methods

### 2.1 Search strategy

A systematic search was conducted across Medline, EMBASE, Cochrane Library, and Epistemonikos to identify clinical trials on statin-treatment for CSVD. The search was performed by a trained information specialist in February 2024, using search terms such as “cerebral small vessel diseases,” “leukoaraiosis,” “subcortical infarct,” “lacunar infarct,” “white matter change,” “lipid regulating agent,” “statin,” and “HMG-CoA reductase inhibitor.” The literature search was restricted to studies published in English, Swedish, Norwegian, and Danish. A detailed description of the full search strategy is provided in Supplementary Material 1.

### 2.2 Selection criteria

This systematic review and meta-analysis followed the Preferred Reporting Items for Systematic Reviews and Meta-Analyses (PRISMA) guidelines [11]. Two investigators (D.F. and B.F.) independently screened the titles and abstracts of retrieved studies for inclusion, based on the following pre-specified selection criteria (PICOS): (1) population: older persons examined with brain MRI/CT for evaluation of WMH; (2) intervention: treatment with statins; (3) comparison: age-matched controls receiving placebo; (4) outcome: WMH volume, changes in WMH volume, HR/OR for progression of WMH and (5) study design: RCT, cohort studies with a control group. Articles in full-text were assessed by the same two investigators.

### 2.3 Data extraction and analysis

Two investigators (D.F. and B.F.) independently extracted data and recorded it in a table that included the following study characteristics: first author, year of publication, country, research setting, study design, sample size, patient demographics, randomization, statin treatment and dosage, and WMH volume and progression.

### 2.4 Statistical analysis

The differences in WMH volume and progression between the treatment and placebo groups were analyzed. Meta-analysis was conducted using inverse variance with RevMan version 5.4.1.22 [12], which automatically assigned weights according to the program’s algorithm. Two-sided p-values were reported, and effect estimates were presented as mean difference (MD).

### 2.5 Quality assessment and risk of bias

The quality of the included studies and their risk of bias were independently assessed by two investigators (D.F. and B.F.) using the Center for Evidence-Based Management (CEBM) critical appraisal worksheet tailored for randomized controlled trials [13]. The overall confidence in the effect estimates across studies for each pooled outcome was independently evaluated by the same two reviewers using the Grading of Recommendations, Assessment, Development, and Evaluations (GRADE) tool [14].

### 2.6 Resolution of discrepancies

Any disagreements arising during the study selection process, data extraction, or quality assessment were resolved through consensus between the investigators.

## 3 Results

### 3.1 Literature search

**Fig. 1:**
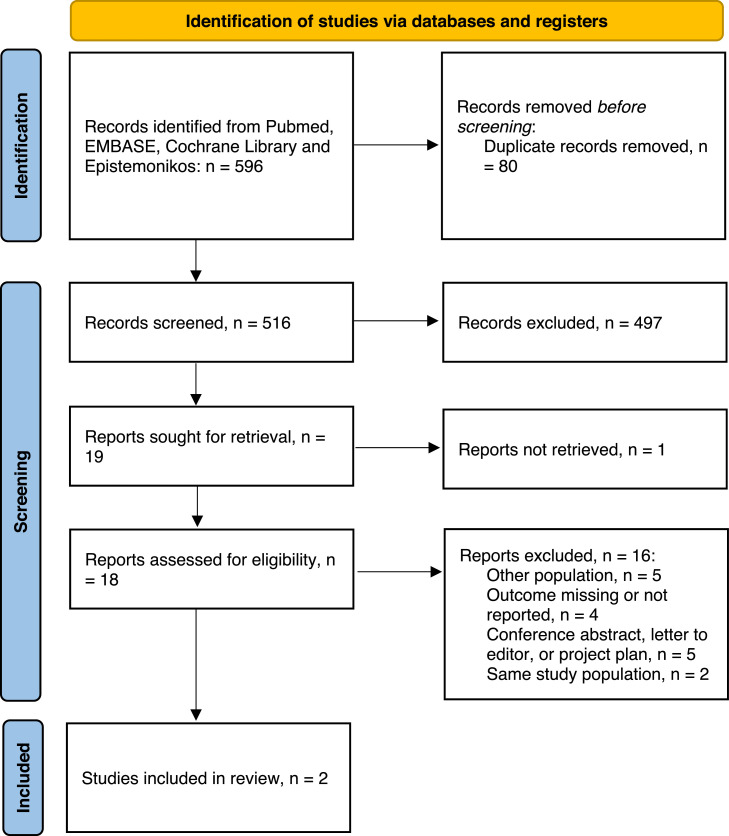
PRISMA flow chart of the inclusion process.

A total of 2 studies [7,15] were included in this review and meta-analysis following a full-text assessment of 18 articles, see Fig. 1. Studies that did not meet the eligibility criteria and were excluded are detailed in Supplementary Material 2.

### 3.2 Study characteristics

The general characteristics of the studies included in this meta-analysis are summarized in Table 1. Included studies were randomized controlled trials published in 2011 and 2020. Criteria used for diagnosis of CSVD were the Fazekas scale, number of lacunes and computer estimated volume of WMH. The types of statins used were rosuvastatin and atorvastatin. Mean age of the research persons across the included studies ranged from 73 to 78 years. WMH burden and Fazekas score are reported in Table 1.

**Table 1:** General study characteristics**.**

Author and year of publication	Guo 2020	Tendolkar 2011
Country	China	Netherlands
Setting	University hospital, community-based sample	Outpatient clinic of university hospital
Sample	Persons with hypertension, ≥75 year	Older-, stroke-free persons with atrial fibrillation
Subjects (n; controls, intervention)	109, 118	17, 17
Criteria for CSVD	STRIVE, Fazekas scale, Lacunes – cavities 3–15 mm with cerebrospinal-like signaling	Manual and computed estimation
Age, mean (SD); controls, intervention	78.48 (2.58), 78.24 (2.35)	73.5 (4.0); 74.5 (4.2)
Sex (male; n (%)	109 (0.48)	17 (50)
Years of education, mean	NR	NR
Statin treatment (substance, dose in mg)	Rosuvastatin, 10 mg/day	Atorvastatin, 20 – 40 mg
Treatment duration in months	61.8	12
Baseline Fazekas score	Prevalence of score of 2 or 3: n, 48; 21.1 %	NR
WMH volume in ml in intervention group at follow-up	Mean 4.29, SD 1.92	Mean 4.19, SD 0.4
WMH volume in ml in controls at follow-up	Mean 5.05, SD 1.91	Mean 11.56, SD 0.01
WMH progression (OR; HR)	0.19, 0.07 – 0.53; 0.408, 0.233 – 0.716)	NR
Progression of lacunar infarcts (OR; HR)	0.34, 0.15 – 0.75; 0.417, 0.257 – 0.676	NR

CSVD, cerebral small vessel disease; HR, Hazard ratio; NR, not reported; OR, odds ratio; SD, standard deviation; STRIVE, Standards for Reporting Vascular Changes on Neuroimaging; WMH, white matter hyperintensities.

### 3.3 Meta-analysis

**Fig. 2:**
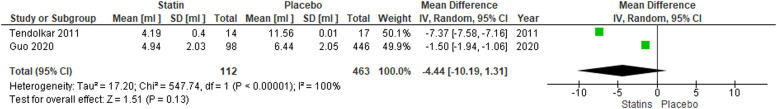
White matter hyperintensities in persons with statins versus no statins.

Data from the included studies with a total of 112 persons treated with statins for CSVD and 463 controls were used for meta-analysis. Sample sizes ranged from 34 [15] to 227 [7]. The total WMH volume was 4.44 ml lower (CI -10.19 – 1.31, *p* = 0.13) in the statin group compared to controls, although not statistically significant, see Fig. 2.

### 3.4 Quality assessment of the included studies

The quality of the included studies was evaluated using the CEBM checklist designed for randomized controlled trials [13], with results presented in Table 2.

**Table 2:** Assessment of overall risk of bias of included studies.

Author and year of publication	Overall appraisal
Guo 2020	+
Tendolkar 2012	+

+, low risk.

### 3.5 Certainty of evidence

The certainty of the pooled estimates was assessed using the GRADE approach, with results presented in Table 3. Our confidence in the pooled estimates was *low* and it is therefore very likely that future studies will have an impact on the results.

**Table 3:** Confidence in the pooled estimate of progression of white matter hyperintensities according to GRADE.

Certainty assessment							№ of patients		Effect		Certainty	Importance
№ of studies	Study design	Risk of bias	Inconsistency	Indirectness	Imprecision	Other considerations	Statin	placebo	Relative (95 % CI)	Absolute (95 % CI)		
**White matter hyperintensity volume in statin vs placebo**												
2	randomised trials	not serious	not serious	not serious	very serious^a^	none	112	463	-	MD **4.44ml lower** (10.19 lower to 1.31 higher)	⨁⨁◯◯ Low	IMPORTANT

CI, confidence interval; MD, mean difference.

a . Small number of studies.

## 4 Discussion

The results from this systematic review and meta-analysis of randomized controlled trials indicate that there is currently insufficient evidence to conclude that statins are effective for treatment of CSVD. Meta-analysis of WMH volume in the two included studies showed a non-significant trend towards a protective effect, although the confidence in the pooled estimate was low according to GRADE. A factor that could attenuate the result is the fact that WMH-burden was markedly higher in the treatment group at baseline in one of the included studies. Only one of the included studies has specifically evaluated the impact of statin therapy on lacunar infarcts, reporting a protective effect [7]. Although the protective effect of statins on cardiovascular events has been demonstrated earlier, this study has examined the current evidence for their effect on cerebrovascular disease in a stroke-free population. One previous meta-analysis from 2020 looked at the effects of statins on WHM-burden in addition to silent brain infarcts, although meta-analysis of the former outcome was not possible due to the limited number of studies available at that time [16].

Rosuvastatin was used in one of the studies and atorvastatin was used in the other. While study results were consistent, it is important to consider that the first mentioned is a water-soluble substance and the latter is lipid-soluble, affecting permeability and accumulation in the central nervous system. If the main effect of statins for the prevention of CSVD is dependent on substance-concentration in the brain, this would therefore be important to consider.

The dose of statins used was low-moderate – 10 mg for rosuvastatin and 20 – 40 mg for atorvastatin; highest recommended dosage is 40 mg and 80 mg, respectively. In this patient group, we have not identified any randomized controlled trials of full-dose statin treatment for comparison.

The exact mechanisms by which statin treatment could reduce CSVD-progression are not yet known. In CSVD related to cardiovascular risk factors, such as hypertension, statins may have a protective effect through several interrelated mechanisms: 1) interfere with the build-up of lipids in blood vessel walls preventing the main pathophysiological contributors – arteriolosclerosis, lipohyalinosis and microatheroma [4]; 2) anti-inflammatory effects through reduction of inflammatory mediators including C-reactive protein [17,18] and interleukin-6 [18]; 3) reduced oxidative stress [19]; 4) protection from microvascular thrombosis by counteracting platelet aggregation and promoting fibrinolysis [20].

Slowing down the CSVD-process may not only reduce the risk of vascular dementia but could also affect the risk of developing Alzheimer’s disease. At present, there is evidence that CSVD may be linked to a higher conversion rate from mild cognitive impairment to Alzheimer’s dementia [21] and a higher risk of developing Alzheimer’s disease in people who are free from cognitive impairment [22]. The mechanisms explaining this association between CSVD and Alzheimer’s disease are not clear, but could be associated to changes in brain fluid drainage, disturbance of the blood-brain barrier or even inflammation processes [23].

Limitations of this systematic review and meta-analysis include: 1) it is based on a small number of studies; 2) even though the literature search was conducted by an experienced information specialist, it is still possible that some articles were missed; 3) due to the risk of imprecision, our confidence in the pooled estimate was rated as low according to GRADE; 4) due to the limited number of studies, no meaningful subgroup analysis of statin-type or dosage was possible; 5) in cases of missing information, authors were contacted although not all of them answered or were able to provide the data necessary. The transparent methodology according to the PRISMA protocol is a strength in this study, as well as the thorough review of available evidence from randomized controlled trials of statin-treatment for prevention of CSVD.

In conclusion, the few studies that have examined the effect of statins on CSVD show a possible protective effect on the brain’s microvasculature. The current evidence is limited and largely based on non-clinical outcomes. The clinical impact of reducing the burden of WMH remains difficult to quantify, particularly given that CSVD progression often coincides with normal physiological aging. For patients and clinicians, it is frustrating that CSVD-related cognitive impairment can continue to progress despite comprehensive treatment with antihypertensive, cholesterol-lowering, antidiabetic, and antiplatelet therapies. Taking this current knowledge gap into consideration, more research is strongly warranted, and future research should focus on clinical and patient-centered outcomes, including global cognitive measures and activities of daily living, alongside surrogate radiological outcomes. Additionally, further randomized controlled trials are needed to investigate the effects of different types and doses of statins on both clinical and radiological manifestations of CSVD.

## Ethical considerations

The data used for this systematic review and meta-analysis has been gathered from previously published literature only. Therefore, application for ethical consent is not necessary as stated in the Swedish law and guidelines of the Ethical Committee of Uppsala University, Sweden.

## Funding

This study was funded by both Örebro University School of Medical Sciences, Örebro, Sweden, and Central Hospital Karlstad, Karlstad, Sweden.

## Generative AI statement

No generative AI tools were used in the conception, writing, or analysis of this scientific paper.

## CRediT authorship contribution statement

**David Fresnais:** Writing – original draft, Validation, Methodology, Formal analysis, Conceptualization, Writing – review & editing, Visualization, Project administration, Investigation, Data curation. **Brynjar Fure:** Writing – review & editing, Validation, Project administration, Investigation, Conceptualization, Visualization, Supervision, Methodology, Data curation.

## Declaration of competing interest

There are no conflicts of interest.

## Appendix Supplementary materials

Image, application 1

Image, application 2
