# Supplementary material for: Statin treatment for cerebral small vessel disease: A systematic review and meta-analysis of randomized controlled trials
Source: Cereb Circ Cogn Behav. 2025 Jun 29;9:100389. doi: 10.1016/j.cccb.2025.100389 (PMC12272895; doi:10.1016/j.cccb.2025.100389)
Supplement: Supplementary file 1 [file mmc1.pdf]

Cerebral small vessel diseases

Ovid MEDLINE(R) ALL <1946 to February 20, 2024>

1	exp Cerebral Small Vessel Diseases/	9961
2	Leukoaraiosis/ or White Matter/ or exp Leukoencephalopathies/	50230
3	("small vessel dis*" or "small vessel cerebrovascular dis*" or SVD or CSVD or "microvascular ischemic dis*" or "cerebral microangiopath*" or "subcortical infarct*" or "subcortical stroke*" or "subcortical lesion*" or "lacunar infarct*" or "lacunar stroke*" or "lacunar lesion*" or "cerebral microbleed*" or "cerebral microhemorrhage" or "cerebral microhaemorrhage" or "cerebral amyloid angiopath*" or "hypertensive angiopath*" or "deep perforator arteriopath*" or "hypertensive arteriopath*" or arteriolosclerosis or "white matter dis*" or "white matter hyperintens*" or "white matter lesion*" or "White matter change*" or "White matter damag*" or "microvascular ischemia" or WML or WMH or WMC or "leukoaraios*" or "Leukoencephalopathy").ab,ti,kf.	41376
4	1 or 2 or 3	87734
5	exp Lipid Regulating Agents/ or exp Hypolipidemic Agents/	166287
6	exp Hypercholesterolemia/ or exp Hyperlipidemias/	71350
7	(statin* or "HMG-CoA reductase inhibit*" or "lipid-lowering*" or "cholesterol-lowering*" or "cholesterol synthesis inhib*" or "cholesterol reduc*" or "Hypolipidemic Agent*" or "anticholesteremic agent*" or Hydroxymethylglutaryl* or "cholesterol absorption inhibitor*" or "PCSK9 inhibitor*" or "proprotein convertase subtilisin/kexin type 9 inhibit*").ab,ti,kf.	77289
8	(antilipemic* or atorvastatin or fluvastatin or lovastatin or pravastatin or rosuvastatin or simvastatin or lipitor or lescol or mevacor or pravachol or crestor or zocor or ezetimibe or fibrate* or alirocumab or evolocumab or praluent or repatha or fenofibrate or gemfibrozil or probucol or "ezetimibe simvastatin" or "bile acid Sequestrant*").ab,ti,kf.	42303
9	Fluvastatin/ or Atorvastatin/ or Probucol/ or exp Lovastatin/ or Pravastatin/ or Rosuvastatin Calcium/ or exp Ezetimibe/ or Fenofibrate/ or Gemfibrozil/ or exp PCSK9 Inhibitors/ or exp Fibric Acids/ or Cholestyramine Resin/ or alirocumab.nm. or evolocumab.nm.	39999
10	5 or 6 or 7 or 8 or 9	259004
11	4 and 10	861
12	limit 11 to clinical trial, all	60
13	limit 11 to randomized controlled trial	32
14	((randomized controlled trial or controlled clinical trial).pt. or randomized.ab. or placebo.ab. or clinical trials as topic.sh. or randomly.ab. or trial.ti.) not (exp animals/ not humans.sh.)	1456876
15	11 and 14	83
16	12 or 13 or 15	101
17	limit 16 to yr="1980 -Current"	101
18	limit 17 to (danish or english or norwegian or swedish)	93

Embase <1974 to 2024 February 20>

1	leukoaraiosis/ or white matter/ or exp leukoencephalopathy/	92955
2	("small vessel dis*" or "small vessel cerebrovascular dis*" or SVD or CSVD or "microvascular ischemic dis*" or "cerebral microangiopath*" or "subcortical infarct*" or "subcortical stroke*" or "subcortical lesion*" or "lacunar infarct*" or "lacunar stroke*" or "lacunar lesion*" or "cerebral microbleed*" or "cerebral microhemorrhage" or "cerebral microhaemorrhage" or "cerebral amyloid angiopath*" or "hypertensive angiopath*" or "deep perforator arteriopath*" or "hypertensive arteriopath*" or arteriolosclerosis or "white matter dis*" or "white matter hyperintens*" or "white matter lesion*" or "White matter change*" or "White matter damag*" or "microvascular ischemia" or WML or WMH or WMC or "leukoaraios*" or "Leukoencephalopathy").ab,ti,kf.	64471
3	1 or 2	132631
4	exp agents affecting lipid metabolism/ or exp antilipemic agent/	445854
5	exp hypercholesterolemia/ or exp hyperlipidemia/	198593
6	(statin* or "HMG-CoA reductase inhibit*" or "lipid-lowering*" or "cholesterol-lowering*" or "cholesterol synthesis inhib*" or "cholesterol reduc*" or "Hypolipidemic Agent*" or "anticholesteremic agent*" or Hydroxymethylglutaryl* or "cholesterol absorption inhibitor*" or "PCSK9 inhibitor*" or "proprotein convertase subtilisin/kexin type 9 inhibit*").ab,ti,kf.	123090
7	(antilipemic* or atorvastatin or fluvastatin or lovastatin or pravastatin or rosuvastatin or simvastatin or lipitor or lescol or mevacor or pravachol or crestor or zocor or ezetimibe or fibrate* or alirocumab or evolocumab or praluent or repatha or fenofibrate or gemfibrozil or probucol or "ezetimibe simvastatin" or "bile acid Sequestrant*").ab,ti,kf.	65293
8	fluvastatin/ or atorvastatin/ or probucol/ or mevinolin/ or pravastatin/ or rosuvastatin/ or ezetimibe/ or fenofibrate/ or gemfibrozil/ or exp PCSK9 inhibitor/ or exp fibric acid derivative/ or colestyramine/ or alirocumab/ or evolocumab/	125115
9	4 or 5 or 6 or 7 or 8	618367
10	3 and 9	3621
11	limit 10 to (clinical trial or randomized controlled trial or controlled clinical trial or multicenter study or phase 1 clinical trial or phase 2 clinical trial or phase 3 clinical trial or phase 4 clinical trial)	337
12	((randomized controlled trial or controlled clinical trial).ab,ti,kf. or ((randomized or placebo).ab. or "clinical trial (topic)"/ or randomly.ab. or trial.ti.)) not (animal experiment/ not (human experiment/ or human/))	1728650
13	10 and 12	228
14	11 or 13	445
15	limit 14 to yr="1980 -Current"	445
16	limit 15 to (danish or english or norwegian or swedish)	437

APA PsycInfo <1806 to February Week 3 2024>

1	cerebral small vessel disease/	599
2	leukoaraiosis/ or white matter/ or leukoencephalopathy/	12576
3	("small vessel dis*" or "small vessel cerebrovascular dis*" or SVD or CSVD or "microvascular ischemic dis*" or "cerebral microangiopath*" or "subcortical infarct*" or "subcortical stroke*" or "subcortical lesion*" or "lacunar infarct*" or "lacunar stroke*" or "lacunar lesion*" or "cerebral microbleed*" or "cerebral microhemorrhage" or "cerebral microhaemorrhage" or "cerebral amyloid angiopath*" or "hypertensive angiopath*" or "deep perforator arteriopath*" or "hypertensive arteriopath*" or arteriolosclerosis or "white matter dis*" or "white matter hyperintens*" or "white matter lesion*" or "White matter change*" or "White matter damag*" or "microvascular ischemia" or WML or WMH or WMC or "leukoaraios*" or "Leukoencephalopathy").ab,ti,id.	10351
4	1 or 2 or 3	18712
5	lipid metabolism disorders/	329
6	exp Lipids/ or exp Statins/	12824
7	(Lipid Regulating Agents or Hypolipidemic Agents or Hypercholesterolemia or Hyperlipidemias).ab,ti,id.	870
8	(statin* or "HMG-CoA reductase inhibit*" or "lipid-lowering*" or "cholesterol-lowering*" or "cholesterol synthesis inhib*" or "cholesterol reduc*" or "Hypolipidemic Agent*" or "anticholesteremic agent*" or Hydroxymethylglutaryl* or "cholesterol absorption inhibitor*" or "PCSK9 inhibitor*" or "proprotein convertase subtilisin/kexin type 9 inhibit*").ab,ti,id.	6467
9	(antilipemic* or atorvastatin or fluvastatin or lovastatin or pravastatin or rosuvastatin or simvastatin or lipitor or lescol or mevacor or pravachol or crestor or zocor or ezetimibe or fibrate* or alirocumab or evolocumab or praluent or repatha or fenofibrate or gemfibrozil or probucol or "ezetimibe simvastatin" or "bile acid Sequestrant*").ab,ti,id.	833
10	5 or 6 or 7 or 8 or 9	19636
11	4 and 10	206
12	limit 11 to ("0200 clinical case study" or "0300 clinical trial")	22
13	(randomized controlled trial or controlled clinical trial).ab,ti. or randomized.ab. or placebo.ab. or clinical trials.sh. or randomly.ab. or trial.ti.	209454
14	11 and 13	10
15	12 or 14	28
16	limit 15 to yr="1980 -Current"	28
17	limit 16 to (danish or english or norwegian or swedish)	28

Epistemonikos 2024-02-22

1	"small vessel dis*" OR "small vessel cerebrovascular dis*" OR SVD OR CSVD OR "microvascular ischemic dis*" OR "cerebral microangiopath*" OR "subcortical infarct*" OR "subcortical stroke*" OR "subcortical lesion*" OR "lacunar infarct*" OR "lacunar stroke*" OR "lacunar lesion*" OR "cerebral microbleed*" OR "cerebral microhemorrhage" OR "cerebral microhaemorrhage" OR "cerebral amyloid angiopath*" or "hypertensive angiopath*" or "deep perforator arteriopath*" OR "hypertensive arteriopath*" OR arteriolosclerosis OR "white matter dis*" OR "white matter hyperintens*" OR "white matter lesion*" OR "White matter change*" OR "White matter damag*" OR "microvascular ischemia" OR WML OR WMH OR WMC OR "leukoaraios*" OR "Leukoencephalopathy"	
2	statin* OR "HMG-CoA reductase inhibit*" OR "lipid-lowering*" OR "cholesterol-lowering*" OR "cholesterol synthesis inhib*" OR "cholesterol reduc*" OR "Hypolipidemic Agent*" OR "anticholesteremic agent*" OR Hydroxymethylglutaryl* OR "cholesterol absorption inhibitor*" OR "PCSK9 inhibitor*" OR "proprotein convertase subtilisin/kexin type 9 inhibit*" OR antilipemic* OR atorvastatin OR fluvastatin OR lovastatin OR pravastatin OR rosuvastatin OR simvastatin OR lipitor OR lescol OR mevacor OR pravachol OR crestor OR zocor OR ezetimibe OR fibrate* OR alirocumab OR evolocumab OR praluent OR repatha OR fenofibrate OR gemfibrozil OR probucol OR "ezetimibe simvastatin" OR "bile acid Sequestrant*" OR alirocumab OR evolocumab	
3	1 AND 2	38
