# Supplementary material for: Statin treatment for cerebral small vessel disease: A systematic review and meta-analysis of randomized controlled trials
Source: Cereb Circ Cogn Behav. 2025 Jun 29;9:100389. doi: 10.1016/j.cccb.2025.100389 (PMC12272895; doi:10.1016/j.cccb.2025.100389)
Supplement: Supplementary file 2 [file mmc2.docx]

**Supplementary material 2.** Excluded articles after full-text review and reasons for exclusion.

| **Excluded articles** | |
| --- | --- |
| **Author and year of publication** | **Reason for exclusion** |
| **Amarenco 2006** [1]  **Bettcher 2017** [2]  **Chen 2018** [3]  **Fukuma 2015** [4]  **Harding 2023** [5]  **Heo 2017** [6]  **Lavallée 2009** [7]  **Lazashvili 2016**  **Mok 2009** [8]  **Niruban 2009** [9]  **Park 2017** [10]  **Tiantian Ji 2018** [11]  **Vogt 2021** [12]  **Williamsson 2014** [13]  **Zhang 2019** [14] | Stroke population  Missing outcome  Conference abstract  Conference abstract  Project plan  Stroke population  Stroke population  Missing outcome  Missing outcome  Letter to editor  Conference abstract  Same study population  Missing outcome  Diabetes population  Same study population |

**References**

1. Amarenco P, Bogousslavsky J, Callahan A, Goldstein LB, Hennerici M, Rudolph AE, et al. High-dose atorvastatin after stroke or transient ischemic attack. N Engl J Med. 2006 Aug 10;355(6):549–59.

2. Bettcher BM, Ard MC, Reed BR, Benitez A, Simmons A, Larson EB, et al. Association between cholesterol exposure and neuropathological findings: The ACT Study. J Alzheimers Dis. 2017;59(4):1307–15.

3. Chen Y, Lu F, Zhang H, Liu Z. A4144 The effect of telmisartan combined with rosuvastatin on the white matter lesions in elderly hypertensive patients. Journal of Hypertension. 2018 Oct;36:e294.

4. Fukuma K. Abstract T P65: Early Statin Intervention Can Reduce the Early Neurological Deterioration and Recurrence in Acute Lacunar Stroke. Stroke [Internet]. 2015 Feb [cited 2025 Mar 6];46(suppl_1). Available from: https://www.ahajournals.org/doi/10.1161/str.46.suppl_1.tp65

5. Harding IH, Ryan J, Heritier S, Spark S, Flanagan Z, McIntyre R, et al. STAREE-Mind Imaging Study: a randomised placebo-controlled trial of atorvastatin for prevention of cerebrovascular decline and neurodegeneration in older individuals. BMJ Neurol Open. 2023;5(2):e000541.

6. Heo SH, Cho AH, Park JH, Lee JS, Kwon HS, Lee MH, et al. Effect of probucol on the progression of white matter hyperintensities in ischemic stroke patients with a higher burden of small vessel disease: the picasso mri-substudy. Journal of the Neurological Sciences. 2017 Oct 15;381:164.

7. Lavallée PC, Labreuche J, Gongora-Rivera F, Jaramillo A, Brenner D, Klein IF, et al. Placebo-Controlled Trial of High-Dose Atorvastatin in Patients With Severe Cerebral Small Vessel Disease. Stroke. 2009 May;40(5):1721–8.

8. Mok VCT, Lam WWM, Fan YH, Wong A, Ng PW, Tsoi TH, et al. Effects of statins on the progression of cerebral white matter lesion. J Neurol. 2009 May 1;256(5):750–7.

9. Niruban A, Myint PK, Potter JF. Placebo-Controlled Trial of High-Dose Atorvastatin in Patients With Severe Cerebral Small Vessel Disease. Stroke. 2009 Sep;40(9):e542–e542.

10. Park H, Kang HG, Cheong JS, Lee H. Antioxidative activity after statin treatment in ischemic stroke patients according to white matter hperintensity. Journal of the Neurological Sciences. 2017 Oct 15;381:979.

11. Ji T, Zhao Y, Wang J, Cui Y, Duan D, Chai Q, et al. Effect of Low-Dose Statins and Apolipoprotein E Genotype on Cerebral Small Vessel Disease in Older Hypertensive Patients: A Subgroup Analysis of a Randomized Clinical Trial. J Am Med Dir Assoc. 2018 Nov;19(11):995-1002.e4.

12. Vogt NM, Hunt JFV, Ma Y, Van Hulle CA, Adluru N, Chappell RJ, et al. Effects of simvastatin on white matter integrity in healthy middle-aged adults. Ann Clin Transl Neurol. 2021 Aug;8(8):1656–67.

13. Williamson JD, Launer LJ, Bryan RN, Coker LH, Lazar RM, Gerstein HC, et al. Cognitive Function and Brain Structure in Persons With Type 2 Diabetes Mellitus After Intensive Lowering of Blood Pressure and Lipid Levels: A Randomized Clinical Trial. JAMA Intern Med. 2014 Mar 1;174(3):324.

14. Zhang H, Cui Y, Zhao Y, Dong Y, Duan D, Wang J, et al. Effects of sartans and low-dose statins on cerebral white matter hyperintensities and cognitive function in older patients with hypertension: a randomized, double-blind and placebo-controlled clinical trial. Hypertens Res. 2019 May;42(5):717–29.
